# Effects of Corroded and Non-Corroded Biodegradable Mg and Mg Alloys on Viability, Morphology and Differentiation of MC3T3-E1 Cells Elicited by Direct Cell/Material Interaction

**DOI:** 10.1371/journal.pone.0159879

**Published:** 2016-07-26

**Authors:** Sepideh Mostofi, Ehsan Bonyadi Rad, Helmar Wiltsche, Ulrike Fasching, Gabor Szakacs, Claudia Ramskogler, Sriveena Srinivasaiah, Muammer Ueçal, Regine Willumeit, Annelie-Martina Weinberg, Ute Schaefer

**Affiliations:** 1 Department of Orthopedics and Orthopedic Surgery, Medical University of Graz, 8036 Graz, Austria; 2 Institute of Analytical Chemistry and Food Chemistry, Graz University of Technology, 8010 Graz, Austria; 3 Research Unit Experimental Neurotraumatology, Department of Neurosurgery, Medical University Graz, 8036 Graz, Austria; 4 Helmholtz-Zentrum Geesthacht, Institute of Material Research, Geesthacht, Germany; 5 Institute of Materials Science and Welding; Graz University of Technology, 8010 Graz, Austria; University of North Texas, UNITED STATES

## Abstract

This study investigated the effect of biodegradable Mg and Mg alloys on selected properties of MC3T3-E1 cells elicited by direct cell/material interaction. The chemical composition and morphology of the surface of Mg and Mg based alloys (Mg2Ag and Mg10Gd) were analysed by scanning electron microscopy (SEM) and EDX, following corrosion in cell culture medium for 1, 2, 3 and 8 days. The most pronounced difference in surface morphology, namely crystal formation, was observed when Pure Mg and Mg2Ag were immersed in cell medium for 8 days, and was associated with an increase in atomic % of oxygen and a decrease of surface calcium and phosphorous. Crystal formation on the surface of Mg10Gd was, in contrast, negligible at all time points. Time-dependent changes in oxygen, calcium and phosphorous surface content were furthermore not observed for Mg10Gd. MC3T3-E1 cell viability was reduced by culture on the surfaces of corroded Mg, Mg2Ag and Mg10Gd in a corrosion time-independent manner. Cells did not survive when cultured on 3 day pre-corroded Pure Mg and Mg2Ag, indicating crystal formation to be particular detrimental in this regard. Cell viability was not affected when cells were cultured on non-corroded Mg and Mg alloys for up to 12 days. These results suggest that corrosion associated changes in surface morphology and chemical composition significantly hamper cell viability and, thus, that non-corroded surfaces are more conducive to cell survival. An analysis of the differentiation potential of MC3T3-E1 cells cultured on non-corroded samples based on measurement of Collagen I and Runx2 expression, revealed a down-regulation of these markers within the first 6 days following cell seeding on all samples, despite persistent survival and proliferation. Cells cultured on Mg10Gd, however, exhibited a pronounced upregulation of collagen I and Runx2 between days 8 and 12, indicating an enhancement of osteointegration by this alloy that could be valuable for *in vivo* orthopedic applications.

## Introduction

The mechanical properties [1–3] and biocompatibility of Mg based implants [4–19] render these more suitable for orthopaedic interventions than implants manufactured using traditional biomaterials such as stainless steel [20,21], cobalt–chromium-based alloys [22–24], titanium and titanium alloys [25,26]. Mg-based implants are, moreover, bioresorbable, and thus offer the potential to treat load-bearing bone fractures without the need for secondary surgery for implant removal, particularly in children [1]. Whilst numerous reports underline the excellent biocompatibility of Mg and Mg alloys when used as orthopaedic implants [5] or vascular stents [27,28], their interaction with bone tissue, and their osteoconductive properties are, to some extent at least, dependent on the corrosion and degradation in the physiological environment of the body and the associated changes to the material surface [29,30]. Pure Mg degradation is associated with the release of gaseous H_2_ and the formation of gas-based bone cavities after implantation [29–33] that might interfere with material-cell interaction and subsequent bone growth and healing [34]. The corrosion of Mg-based implants is, furthermore, associated with increased pH and the release of ions into the surrounding medium [35,36] which creates an alkaline, hypertrophic environment that can negatively influence cellular activities such as cell attachment, proliferation, differentiation and, ultimately tissue formation [18,37,38]. Morphological features and the chemical composition of the corroded surfaces can moreover modulate cell characteristics during material-cell interaction, though in a manner less studied to date [39–41]. Mg and Mg-based alloy bone implants should ideally provide a platform at the implant interface that promotes tissue regeneration [42,43]. To this end, in order to facilitate initial material-cell interaction and subsequent cell growth and differentiation, conditions that facilitate material-cell interaction and induce tissue regeneration must be established [44,45]. An understanding of the processes and material changes that might have detrimental effects on the cells at the bone-implant interface is a prerequisite to the controlling of material-cell interaction [46–54].

The aim of the present study was to analyse the surface morphology and/or the chemical surface elements associated with the corrosion of Pure Mg, Mg2Ag and Mg10Gd by a physiologically relevant cell medium and the subsequent effects on selected cell properties. Mg alloys containing small quantities of rare earth elements, zinc, thorium or silver, have been proven to possess advantageous mechanical properties and corrosion characteristics [33,55]. The use of gadolinium as a highly soluble rare earth element in an experimental Mg alloy system has been reported to improve both of these features [56–59]. The biocompatibility of this material has, however, not as yet been widely studied [6,39]. Heat treated Mg2Ag alloys have been reported to exhibit better mechanical stability than Pure Mg [60]. Mg2Ag implants have, moreover, been shown to support human osteoblast adhesion and viability. There is also evidence that an increased atomic ratio of silver in Mg2Ag alloys increases antimicrobial activity, and thus the degree to which the implant itself can help to control contamination during, and after bone surgery [60].

Biocompatibility was analysed in the present study by directly growing MC3T3-E1 cells on Pure Mg and Mg alloy samples. MC3T3-E1 cells were originally established from the calvaria of an C57BL/6 mouse embryo/foetus, and have been shown to differentiate into osteoblasts and to produce collagen [61].

A comparison of the interaction of MC3T3-E1 cells with corroded and non-corroded Mg and Mg-based alloy surfaces could potentially provide information enabling the rational design of pre-treatments that improve the biocompatibility of Mg based implants for orthopaedic interventions.

## Materials and Methods

### Materials and sample preparation

Two Mg alloys Mg2Ag (1.75% Ag, Mg Bal*), Mg10Gd (10.5% Gd, Mg Bal.) and Pure Mg (99.99% Mg) were used in our study. Ag and Gd concentration were determined by X-ray fluorescence spectrometry according to weight percentage (Bruker AXS S4 Explorer, Bruker AXS GmbH., Germany). Materials were cast at HZG-MagIC (Geesthacht, Germany) (Table 1).

**Table 1:** Composition of alloying elements in Magnesium-based implants.

Alloy	Composition wt. %					
	Ag	Gd	Fe	Cu	Ni	Mg
Mg	-	-	0.0055	0.003	0.0018	Bal.
Mg10Gd	-	10.5	-	-	-	Bal.
Mg2Ag	1.75	-	0.0022	0.002	0.0013	Bal.

Pure materials were used for casting: Mg (99.99%, XINXIANG JIULI MAGNESIUM CO. LTD, China), Gd (99.95%, Grirem Advanced Materials Co., Ltd., China) and Ag (99.99%, ESG Edelmetall-Handel GmbH & Co. KG, Germany). Mg2Ag and Mg10Gd were produced by permanent mould gravity casting. Pure Mg was melted at 720°C and preheated elements (Ag, Gd) added with continuous stirring. The melt was poured into a preheated (550°C) permanent steel mould treated with Boron Nitride. Cover gas was used during the casting process (SF_6_ and Argon mixture). Solution treatment was applied to the alloys for 16hr at 440°C under argon atmosphere (T4 treatment) [60]. Alloys were homogenized with a T4 heat treatment prior to extrusion in an Argon atmosphere at 550°C (Mg10Gd) and at 420°C (Mg2Ag) for 6hr, and afterwards indirectly extruded with an extrusion ratio of 4/25. The extrusion machine chamber temperature was set at 370°C and the billets (d = 30 mm) preheated for 1hr at 370°C (Mg2Ag) and at 430°C (Mg10Gd). Extrusion was performed at speeds of 3 and 4.5 mm/s. Pure Mg was cast by permanent mould direct chill casting. The cast billet (d = 110 mm) was extruded indirectly with an extrusion ratio of 1/84. Billet temperature was 340°C and extrusion speed 0.7 mm/s. Discs (diameter 10 mm and thickness 1.5 mm) were machined from the extruded bars.

### Surface Analysis

Surface topography and roughness parameters were measured with a Contour GT—K Bruker Profilometer using white light interferometry. Stitching was performed with 5X magnification and an overlap of 10%. The range between back-scan and length was set at -150 μm to + 150 μm. Autoscan was enabled to stop every stitch after another 20 μm measurement, after collection of 90% of the data. A threshold of 1% was used to increase the pixel number. The software program “Vision 5.41” was utilized for measurement data preparation. The applied F-Operator “cylinder and tilt” removed the nominal form of the surface. A total of 3 samples (diameter of area of interest 9.5 mm) were measured.

Samples for scanning electron microscopy were embedded in epoxy resin, ground with 220–2500 grit SiC paper and polished successively with a 3 μm diamond suspension and a 1 μm diamond/OPSTM anhydrous suspension mixture. Samples were etched in an etchant (140 mL ethanol, 30 mL deionized water, 7 mL glacial acetic acid and 8 g picric acid), washed with ethanol and blow-dried with hot air. Microstructures were observed using an optical microscope (Reichert-Jung MeF3) equipped with a digital camera. Grain size was determined using the line intercept method to calculate the middle square grain diameter.

The above treatments generated alloys with comparable surface topography and roughness parameters (S1A and S1B Fig). Slight differences between them were not statistically significant. Analysis of microstructure and grain sizes furthermore revealed that all three alloys were of the same magnitude (middle square grain diameter was 38 ±1 μm, 40 ±1 μm and 27 ±1 μm for Pure Mg, Mg2Ag and Mg10Gd respectively) (S2 Fig).

### Corrosion assay of Mg and Mg based alloys

Disc samples with a diameter of 10 mm and thickness of 1.5 mm were sterilized using ץ-ray irradiation (25 kGy). All samples were cleaned for 10 min in ethanol (70%) and air dried. Corrosion was induced by sample incubation in 4 mL Dulbecco's Modified Eagle's Medium (Invitrogen, Carlsbad, CA, USA) supplemented with 10% foetal bovine serum (Sigma-Aldrich, St. Louis, MO, USA) for 1, 2, 3 and 8 days without changing the medium during immersion time. Corrosion profiles were assessed by measurement of Mg ion concentration in the supernatants at the specified time points. Supernatant of the same samples (with three days of immersion) were used to evaluate the cell viability. Dilutions were assessed based on the highest Mg^2+^ concentration observed after 3 days of immersion. In order to measure the Mg^2+^ concentration, supernatants were centrifuged for 10 minutes at 4500 rpm. Mg concentrations were determined using a simultaneous, axially viewed inductively coupled plasma optical emission spectrometer (ICP-OES; Ciros Vision EOP, Spectro, Germany) equipped with a cross-flow nebulizer, a Scott type PFA spray chamber and a standard ICP torch with a 2.5 mm injector diameter. The selected plasma conditions were 1350 W RF power, 12.5 L/min outer gas flow, 0.6 L/min intermediate gas flow and 0.83 L/min nebulizer gas flow. The 280.270 nm Mg emission lines were used. Calibration standards (0–10 mg/L) were prepared from a multi-element stock solution (100 mg/L, 28 element stock solutions, Roth, Germany). A 1 mg/L internal standard (Sc) was used to compensate for instrument drift. Medium without samples were used as control. Mg ion release was also analysed following culturing of MC3T3-E1 cells on the samples. MC3T3-E1 cells were cultured on non-corroded Pure Mg, Mg2Ag and Mg10Gd for 1, 2 and 3 days, and the level of Mg ion release compared to samples without cells.

The surface composition of corroded and non-corroded samples was analysed using a LEO 1450VP scanning electron microscope (ZEISS, Oberkochen, Germany) equipped with an EDS detector (BRUKER QUANTAX 400) and with an accelerating voltage of 15 kV. The Quantifications were performed semi-quantitatively without a standard sample. Corroded and non-corroded samples and the time points described above were also utilized for cell culture experiments.

### Cell culture

Murine MC3T3-E1 pre-osteoblast cells (ECACC, Salisbury, UK; catalogue number 99072810 were utilized for cell culture studies [62]. Cells were cultured in Eagle’s minimum essential medium (Sigma-Aldrich, St. Louis, MO, USA) supplemented with 10% foetal bovine serum (Sigma-Aldrich), 100 U/mL penicillin, 100 μg/mL streptomycin and 2 mM glutamine (all reagents, Invitrogen, Carlsbad, CA, USA) at 37°C, 5%CO_2_ and 95% humidity. MC3T3-E1 cells (5×10^4)^ were seeded onto corroded (see above) and non-corroded Mg and Mg alloy samples. All three materials were immersed in cell culture medium (DMEM supplemented with 10% FBS) for 1, 2 and 3 days, after which times the cell culture medium was removed. The samples were then transferred to a fresh 24 well plate using plastic forceps from the sides without touching the surface of the material. 5x10^4^ cells in 50 μL were immediately added to the surface of the materials. Cells were allowed to adhere for 30 minutes before gentle addition of 2 ml of medium from one side to cover the surface of the material. Cells were cultured for 24 hr on corroded and non-corroded, Mg and Mg alloy samples.

### Live/Dead staining

After cultivation of cells for 24hr on corroded and non-corroded Mg and Mg alloy samples, cell viability was assessed by Live/Dead staining (Invitrogen). Cells were additionally cultivated only on non-corroded samples for 4, 6, 8 and 12 days and the medium was changed every second day. Samples were washed with phosphate buffer solution (PBS, Gibco, Invitrogen) after removal of the culture medium. 5 μL Calcein AM and 20 μL Ethidium homodimer-1 were mixed with 10 mL PBS. 2ml of freshly prepared staining solution was added to each well of the plate. Following incubation for 25 min in the dark at RT, the samples were rinsed with phosphate buffered saline without CaCl_2_ and MgCl_2_ (PBS, Invitrogen). Pictures were taken with an inverted fluorescence microscope (Olympus) and merged using microscope software.

### Rhodamine-phalloidin staining of cytoskeleton

MC3T3-E1 cells cultivated on corroded and non-corroded Mg and Mg alloy samples, were fixed with 4% Paraformaldehyde (Merck, Darmstadt, Germany) for 10 min at RT and permeabilized with 0.3% Triton-X100 (Sigma-Aldrich) for 5 min at RT. A Rhodamine-phalloidin stock solution (Invitrogen) in methanol (Merck) with a concentration of 6.6 μM (300 U/mL) (540/565 nm), was diluted in PBS (5 μL stock in 200 μL PBS) and incubated for 20 min at RT to stain F-actin bundles. Nuclei were visualized by 4',6-Diamidino-2-Phenylindole, Dihydrochloride (DAPI) counterstaining (Invitrogen) for 5 min at RT using a stock solution concentration of 5 mg/mL (10.9 mM) and working solution concentration of 300 nM) (358/461 nm). F-actin arrangement was analysed based on Laser Scan Confocal images (Zeiss LSM 510).

### Scanning electron microscopy

Following fluorescence imaging, cells were fixed in 2% Paraformaldehyde/2.5% Glutaraldehyde in 0.1M Cacodylatebuffer (Fluka BioChemika GmbH, Buchs, Switzerland) for 30 min, and then rinsed with 0.1M Cacodylate buffer (Merck) (pH 7.4) and subjected to progressive alcohol dehydration (Merck) (30%, 50%, 70%, 80%, 90% and 96%, each for 15 min) at RT. Samples were finally twice immersed in 100% ethanol twice for 15 minutes at RT. Following immersion in anhydrous Acetone for 5 minutes, each sample underwent critical point drying with BAL TEC CPD 030 (formerly BAL TEC, now Leica, Germany) and before coating with gold-palladium BAL TEC SCD500 (former BAL TEC, now Leica, Germany) for 60 sec. Pictures were taken using a (Zeiss DSM950) scanning electron microscope with an acceleration voltage of 15kV.

### Indirect cytotoxicity testing (MTT assay)

The effects of corrosion products, particularly Mg ion concentration, on MC3T3-E1 cells were determined by MTT assay (3-(4,5-dimethylthiazol-2-yl)-2,5-diphenyltetrazolium bromide). Cells were incubated with supernatants from the corrosion assays containing Mg ions at concentrations of 0.3, 0.6, 0.9 and 1.2 mg/mL, according to the concentrations established by corrosion analysis. Before adding the extracts to the cells, pH was adjusted to 7.4. Cellular metabolic activity was also checked for the extracts without pH adjustment. MC3T3-E1 cells were seeded into 48-well tissue culture dishes (Costar; Corning, USA) at a density of 4×10^4^ cells per well and incubated for 24hr to allow optimal attachment, after which the medium was replaced by 250 μL of alloy supernatant. After incubating the cells at 37°C, 5% CO_2_ for 24hr, 25 μL MTT (Invitrogen) was added to each well and the samples were incubated with MTT in DMEM for 4hr. 250 μL DMSO solution (Merck) was then added to each well and 100 μL of the supernatant was analysed by spectrophotometry (570 nm) in 96 well-plates (Spectrostar-Ommega). Cells treated with DMEM medium were used as negative control.

### Immunocytochemistry

5×10^4^ cells MC3T3-E1 were seeded on non-corroded Mg10Gd, Pure Mg and Mg2Ag. After incubation for 2, 4, 8 and 12 days at 37°C, 5% CO_2_ in Minimum Essential Medium Eagle(MEM) medium (Sigma-Aldrich) supplemented with 10% FBS (Sigma-Aldrich), samples were washed twice with PBS and fixed with 4% formaldehyde (Merck) for 10 minutes at RT. After washing twice with PBS, the cells were permeabilized with 0.3% Triton X100 (Sigma-Aldrich) in PBS for 5 minutes at RT. The cells were washed three times in PBS. Blocking solution prepared in 0.3% Triton X100 contained 1% Bovine serum albumin (Sigma-Aldrich) and 5% horse serum (Sigma-Aldrich). A rabbit anti-Collagen 1 polyclonal (Catalogue number ab21286; Abcam, Cambridge, UK) was used as a primary antibody at a dilution of 1:100 for 4 hours in RT. Highly cross adsorbed anti-rabbit IgG (H+L) (Sigma-Aldrich) was used as secondary antibody at a 1:200 dilution. Pictures were taken using a laser scanning microscope (Zeiss, LSM 510).

### Western Blot

MC3T3-E1 cells were cultured on non-corroded Mg10Gd, Pure Mg and Mg2Ag at a density of 5×10^4^ cells in Eagle’s minimum essential medium supplemented with 10% foetal bovine serum (FBS) (Sigma-Aldrich), 100 U/mL penicillin, 100 μg/mL streptomycin and 2 mM glutamine (all reagents, Invitrogen, CA, USA) at 37°C, 5% CO_2_ and 95% humidity for 2, 4, 6, 8, 10 and 12 days. Cells were detached by incubation in 2 mL of Accutase (Invitrogen) for 10 minutes (under cell culture condition) followed by addition of 10 mL MEM supplemented with 10% FBS. The cell suspension was centrifuged for 5 min at 1400 rpm and the resultant cell pellet washed twice by addition of 1 mL medium and repeated centrifugation at 4500 rpm (micro centrifuge) for 3 minutes. Whole cell lysates for immunoblotting, (Radio Immunoprecipitation Assay) were prepared using (RIPA) lysis buffer (Sigma). Protein concentrations were measured using the Bradford protein assay (BioRad, Hercules, CA). Protein lysates (5μg/lane) were separated by 10% sodium dodecyl Sulphate polyacrylamide gel electrophoresis (SDS-PAGE) and transferred to polyvinylidene difluoride (PVDF) membranes. After transfer, to avoid stripping, the membrane was cut and probed overnight with a Runx2 (D1L7F) rabbit monoclonal antibody (catalogue number 12556S;Cell Signalling, USA), a rabbit anti-Collagen I polyclonal (catalogue number ab21286; Abcam) and rabbit anti-B-actin (N21) polyclonal antibody (catalogue number sc-130656; Santa Cruz, USA) at dilutions of 1:4000, 1:4000 and 1:500 respectively. A peroxidase-conjugated goat anti-rabbit secondary antibody (catalogue number sc-2054; Santa Cruz, USA) was then added at a dilution of 1:1000. Protein bands were detected using ECL chemiluminescence reagents (Amersham) and exposure to X-ray films. Band densities for the proteins of interest were measured at each time point, and normalized to the density of the house keeping gene (B-Actin). Protein expression levels were subsequently compared to the biological control (Cells cultured on tissue culture plate).

### Statistical analysis

Statistical analyses were performed using SPSS software (SPSS Inc., Chicago, IL, USA). Normal distribution of the samples was determined using the Shapiro-Wilk normality test. The mean values for quantitative data and standard errors for ICP-OES, MTT and Western blot results were analysed by applying the One-Way ANOVA. P value (statistical significance) was set at 0.05.

## Results

### Analysis of surface morphology, atomic % of chemical surface elements and the release profile of Mg ions during sample degradation

Surface morphology, atomic % of chemical surface elements, and the release of Mg ions into the supernatant are factors that might strongly affect the properties of MC3T3-E1 cells cultured on the surface of corroded Mg samples. These parameters were analysed for Pure Mg, Mg2Ag and Mg10Gd, following immersion for 1, 2, 3 and 8 days in DMEM supplemented with 10% FBS.

**Fig 1:**
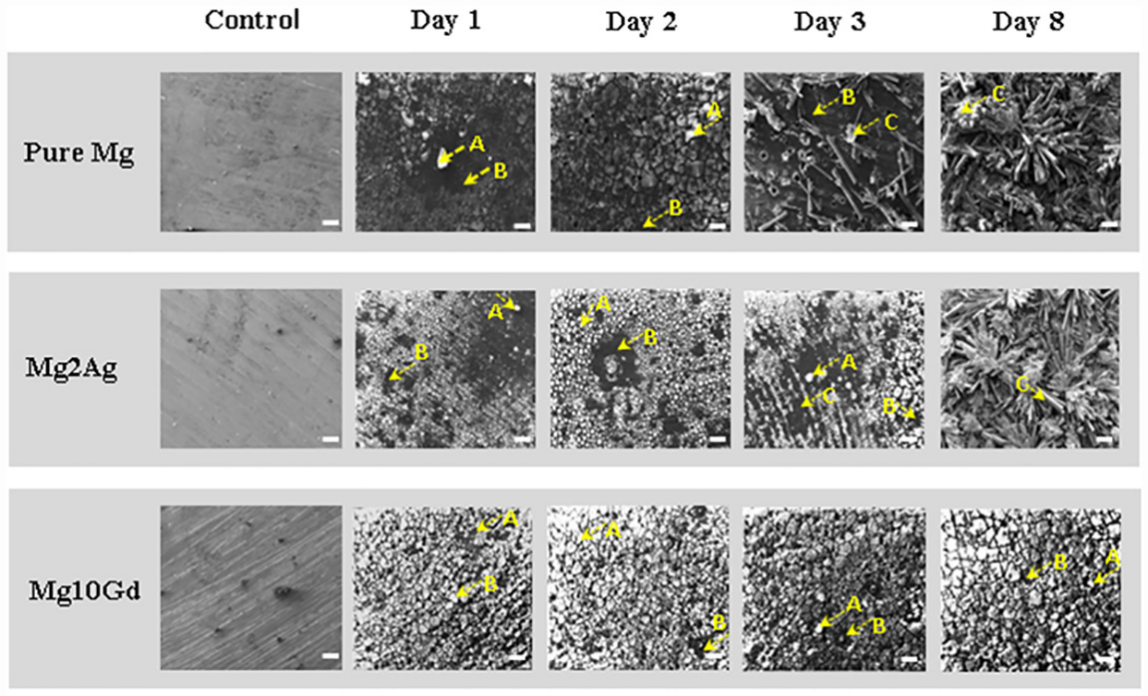
Changes in Surface morphology of Mg and Mg alloys during corrosion at day 1, 2, 3 and 8. Changes in Mg and Mg alloys Surface morphology were determined by scanning electron microscopy at day 1, 2, 3 and 8 following immersion in DMEM with 10% FBS. Two regions were detected during corrosion light seemingly protruding grains (arrows A) and dark areas (arrows B). Crystal formation (arrows C) was detected after 3 and 8 days following immersion on the surface of Mg2Ag and Pure Mg. **Scale bars**: 100 μm.

In scanning electron microscopy images, the surfaces of all samples appeared fissured (riverbed) resulting in region appearing as dark shadows (A) or light seemingly protruding grains (B), indicating corrosion product deposition (Fig 1). Needle-shaped crystals (C) emerged from the surface of Pure Mg and Mg2Ag following a 3 day immersion. Although crystals detected on Mg2Ag were remarkably smaller and can be better seen at higher magnifications. Pure Mg and Mg2Ag were both covered in needle-shaped crystals by day 8. Crystal formation on the surface of Mg10Gd in contrast was negligible.

EDX results showed Mg, O, Ca, P and C to be the major elements on the surface of all three materials, independent of areas (dark shadows, light grains) or structural appearance (crystals) (S3 Fig).

**Fig 2:**
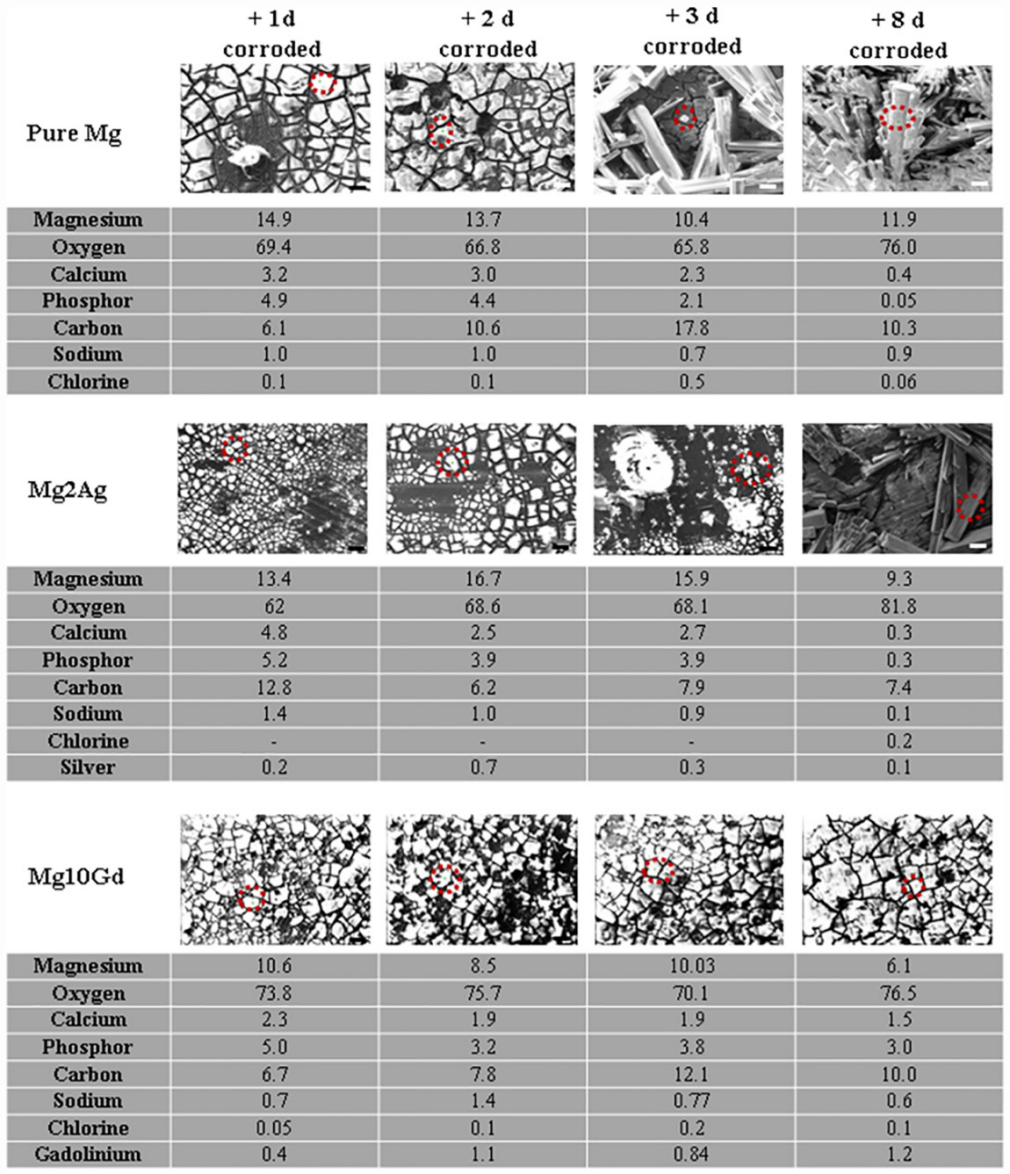
Atomic % of chemical elements on corroded surfaces of Pure Mg, Mg2Ag and Mg10Gd at 1, 2, 3, 8 days of immersion in DMEM with 10% FBS determined by scanning electron microscopy equipped with EDS. Distribution of chemical surface elements on the surface of Pure Mg, Mg2Ag and Mg10Gd were analysed by scanning electron microscopy equipped with EDS. Location of analysis is depicted by red circles. Analysis established atomic percentage of the elements on the surface. **Magnification: 500X**.

The composition of the corrosion deposits (light protruding grains) on Pure Mg and Mg alloys appeared to remain unchanged up to three days following immersion, with a high oxygen content indicative of oxidative corrosion. Crystal appearance was associated with a notable increase in the atomic % of oxygen on the surface of these samples, indicating crystal formation to be associated with enhanced oxidation (Fig 2). Since this effect was observed with both Pure Mg and the Mg2Ag alloy, a negligible effect of Ag on the oxidative corrosion process can be assumed. Time dependent changes in the atomic % of oxygen on the surface of the Mg10Gd alloy were, in contrast, not observed. It should, however, be noted that, at any given time point, the oxygen: Mg ratio on the Mg10Gd surface was higher than on the surface of Pure Mg or Mg2Ag (Table 2). Crystal formation was, thus, not solely determined by the atomic % of oxygen or the oxygen: Mg ratio on the sample surface. Crystal formation on the surface of Pure Mg and Mg2Ag was also linked to a marked (5–10 fold) decrease in the calcium and phosphorous surface content. Phosphorous and/or calcium content was not reduced on the surface of crystal-free Mg10Gd, indicating a role for phosphorous and/or calcium in the inhibition of oxygen associated crystal formation. It is noteworthy in this context that Gd has been reported to chelate phosphorous and calcium [63].

**Table 2:** Oxygen: Mg ratio on the sample surface.

O:Mg Ratio	Day 1	Day 2	Day 3	Day 8
Pure Mg	4.6	4.8	6.3	6.3
Mg2Ag	4.6	4.1	4.2	8.7
Mg10Gd	4.6	8.9	6.9	12.5

Ratio of the Oxygen to Magnesium detected from elemental composition analysis during 1, 2, 3 and 8 days corrosion of Pure Mg, Mg2Ag and Mg10Gd.

Whilst EDX analysis does not allow conclusions to be drawn regarding degradation mechanism(s) or the actual composition of the corrosion deposits (i.e. Mg (OH) _2_) which will be strongly influenced by the DMEM medium content, it does allow surface morphology and, to some extent, chemical surface content to be linked to potential modulations of cell properties.

DMEM is a complex solution including, salts, glucose (mirrored in the surface carbon content), amino acids, and variety of proteins, all of which might be also found *in vivo* in tissue and which are indispensable for cell survival. The precise corrosion mechanisms induced by cell medium, in particular by the rapid surface adhesion of proteins and carbon, therefore, require more detailed analysis.

The properties of MC3T3-E1 cells cultured on the sample surfaces, for instance viability, could also be modulated by corrosion associated release of Mg ions into the supernatant, as well as surface morphology and chemical surface content.

**Fig 3:**
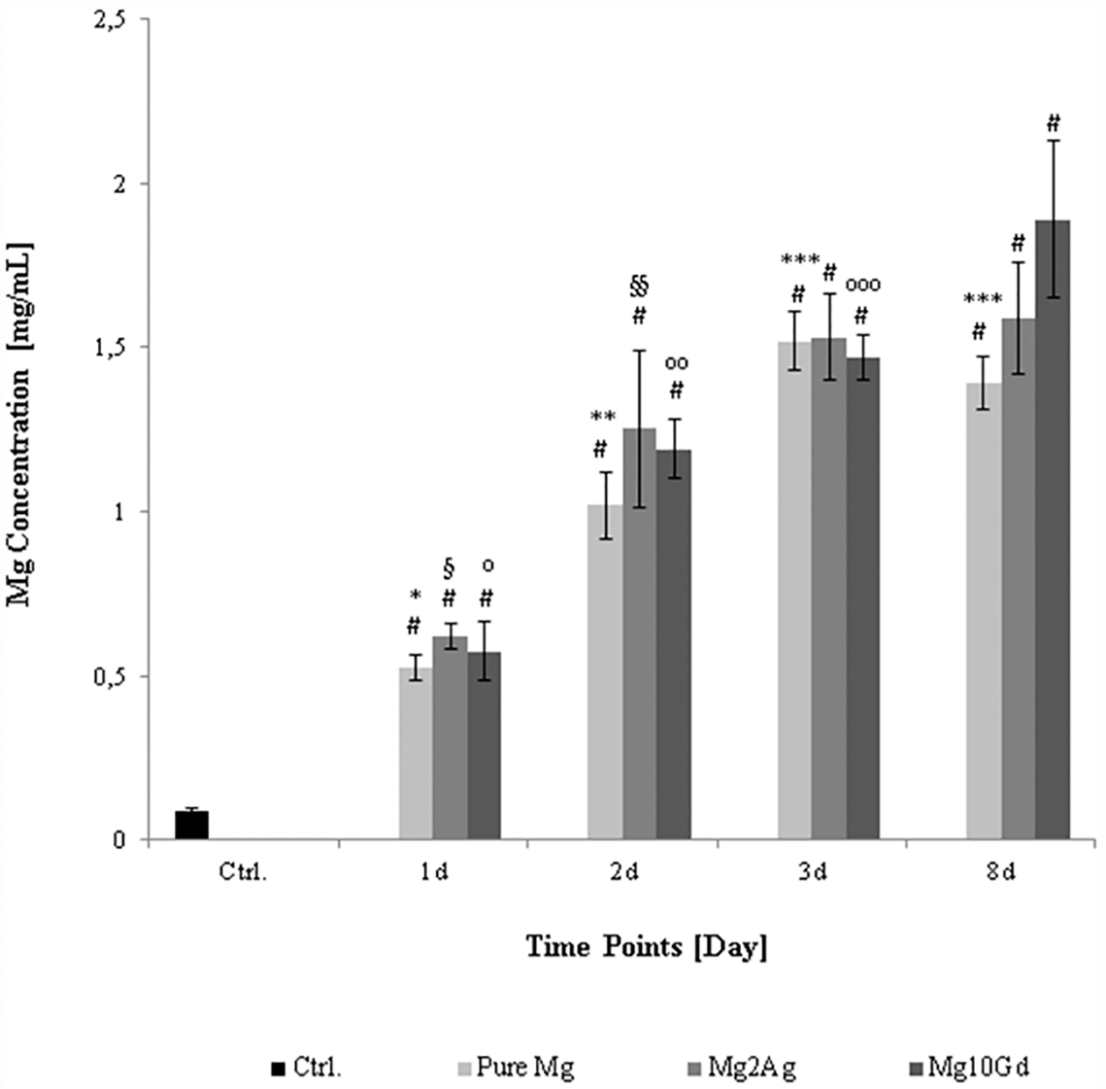
Changes in Mg^2+^ release into the supernatant during corrosion of Pure Mg, Mg2Ag and Mg10Gd determined by ICP-OES analysis. Mg ion concentration in the supernatant (DMEM, 10%FBS) of Mg and Mg alloys was measured at 1, 2, 3 and 8 days with ICP-OES method; n = 5. Statistical significance was tested with One-way ANOVA test. **# p<0.05** as compared to the control (Magnesium level of the basal medium); * p<0.05 as compared to ion release from Pure Mg at day 2, 3 and 8; ** p<0.05 as compared to ion release from Pure Mg at day 1, 3 and 8; *** p<0.05 as compared to ion release from Pure Mg at day 1 and 2; **§ p<0.05** as compared to ion release from Mg2Ag at day 2, 3 and 8; **§§ p<0.05** as compared to ion release from Mg2Ag at day 1 and 8;° **p<0.05** as compared to ion release from Mg10Gd at day 2, 3 and 8;°**° p<0.05** as compared to ion release from Mg10Gd at day 1, 3 and 8;°**°° p<0.05** as compared to ion release from Mg10Gd at day 1, 2 and 8.

A continuous and significant increase of Mg ion release from Pure Mg and Mg2Ag samples was detected during the first 3 days of immersion that plateaued by day 8, indicate of a reduced rate of degradation at this time point (Fig 3). The timing of reduced Mg ion release coincided with that of crystal formation, indicating crystals to be relatively stable corrosion deposits. The rate of Mg ion release by Mg10Gd continuously increased throughout the 8 day immersion period (1.8±0.01 mg/mL), indicative of on-going corrosion, and the formation of less stable corrosion products on this material.

Mg ion release is usually associated with an increase in pH that can also affect the corrosion process [64]. The time dependent increase in pH was, however, minimal in this case (with pH varying between 8.5 and 8.6), and did not differ significantly between the supernatants of Pure Mg, Mg2Ag and Mg10Gd, indicating effective buffering by HEPES buffer in the DMEM medium and by exogenous CO_2_ (S4 Fig).

### Cell Viability and morphology following culturing of MC3T3-E1 cells on corroded and non-corroded Mg samples

**Fig 4:**
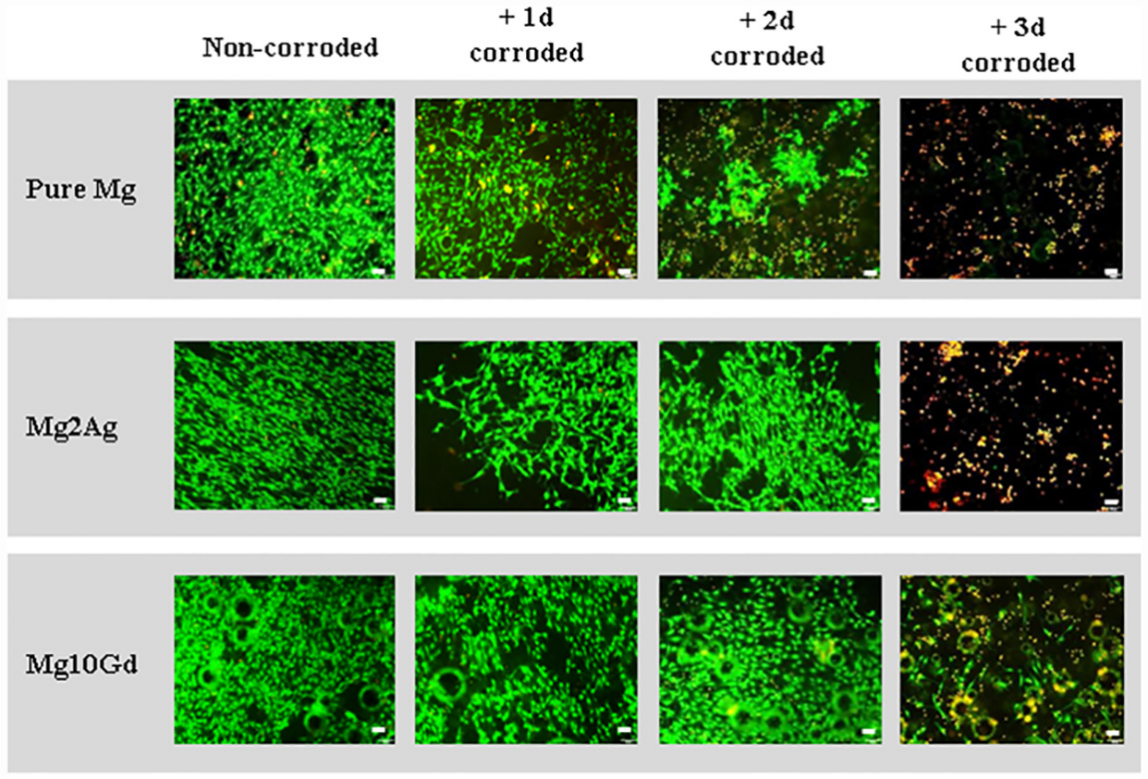
Viability of MC3T3-E1 cells cultured on samples pre-corroded for 1, 2, 3 days and non-corroded Pure Mg, Mg2Ag, Mg10Gd determined by live-dead staining. Viability of MC3T3-E1 cells were evaluated after 24hrs of cultivation on Pure Mg, Mg2Ag and Mg10Gd which were corroded for 1, 2, 3 days. Non-corroded samples were used as a control. **Scale bars**: 100 μm in all the pictures.

Pre-corroded Mg samples generated by immersion in DMEM medium for 1, 2, or 3 days were transferred into culture plates without further manipulation. MC3T3-E1 cells (50,000 cells/50 μL) were adhered to the corroded surface of the samples for 30 min at 37°C in an incubator. 2 ml of fresh medium was then added and the cells were cultured on the surface of the Mg and Mg alloys for 24 hours at 37°C. Control cells were cultured on non-corroded Pure Mg, Mg2Ag and Mg10Gd samples using the same procedure. Viability was determined by Live/Dead staining (Fig 4). A notable time-dependent decrease in viability was observed when MC3T3-E1 cells were cultured on Pure Mg samples pre-corroded for 1 to 2 days, with no detectable survival of cells cultured on Pure Mg samples pre-corroded for 3 days. Survival of MC3T3-E1 cells cultured on pre-corroded Mg2Ag and Mg10Gd was only significantly reduced by samples pre-corroded for 3 days, with greater survival of cells cultured on pre-corroded Mg10Gd. Viability was not significantly affected by culturing MC3T3-E1 cells on non-corroded Mg samples (Fig 4).

**Fig 5:**
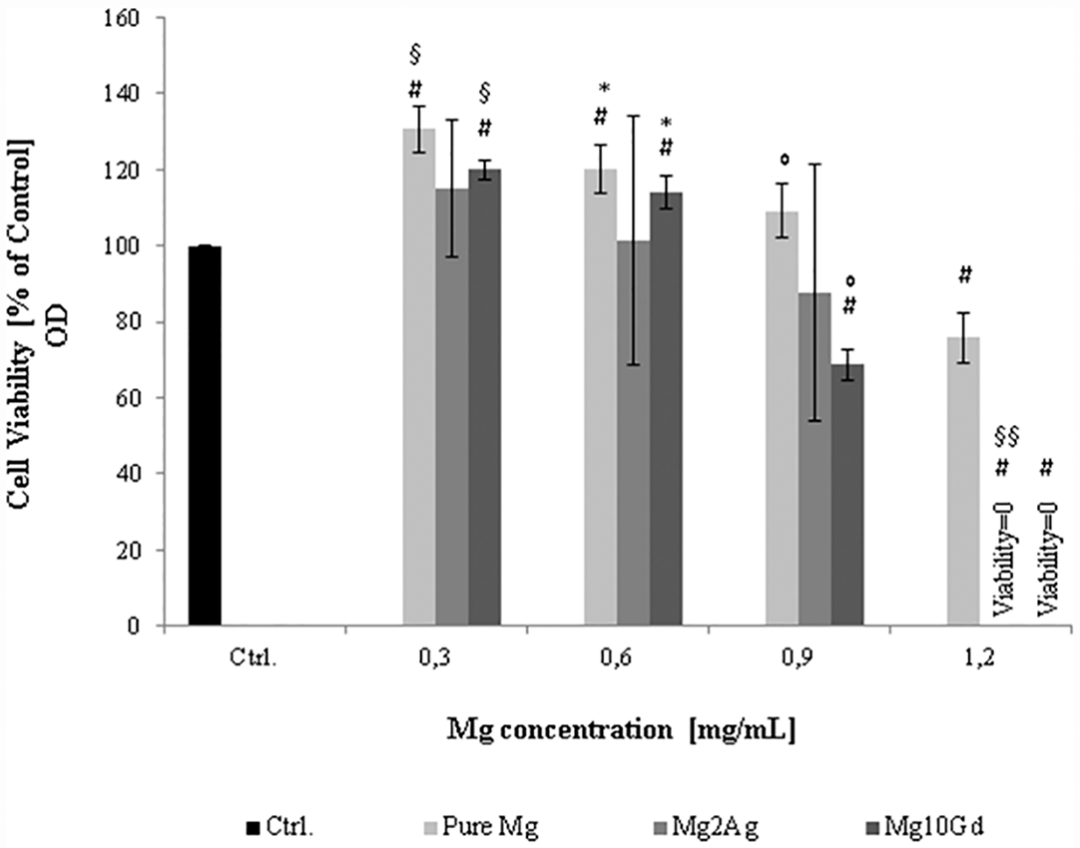
Viability of MC3T3-E1 cells treated with different concentration of Mg^2+^ (diluted to standard concentrations from the supernatant of Pure Mg, Mg2Ag and Mg10Gd). Different concentration of Mg^2+^ (0.3–0.6–0.9 and 1.2 mg/mL) were added to the cells and after 24hrs cell viability were determined by MTT assay. The pH of the extracts has been adjusted to 7.4. Decrease in viability of MC3T3-E1 cells was detected at concentration of 0.9 to 1.2 mg/mL Mg^2+^. Statistical significance was tested with One-way ANOVA test. **# p<0.05** as compared to the cell viability of the control; **§p<0.05** as compared to cell viability at concentration of 0.6, 0.9 and 1.2 mg/mL Mg^2+^ resulted from Pure Mg and Mg10Gd extracts; *p<0.05 as compared to cell viability at concentration of 0.9 and 1.2 mg/mL Mg^2+^ concentration resulted from Pure Mg and Mg10Gd extracts;° **p<0.05** as compared to cell viability at 1.2 mg/mL Mg^2+^ concentration resulted from Pure Mg and Mg10Gd extracts; **§§ p<0.05** as compared to cell viability at concentration of 0.3, 0.6 and 0.9 mg/mL Mg^2+^ concentration resulted from Mg2Ag (Significance level was set at p < 0.05; n = 4 per group).

A possible effect of corrosion-associated Mg ion release on cultured cell viability and/or proliferation was assessed by metabolic analysis (MTT test) of MC3T3-E1 cells following incubation with increasing Mg ion concentrations. Supernatants from the time-dependent corrosion assays (Mg ion concentration presented in Fig 3) were diluted to establish a standardized and matching concentration range (0.3–0.6–0.9–1.2 mg/mL) for this purpose. A significant decrease in metabolic activity, i.e. viability and/or proliferation, was only observed at Mg ion concentrations of 0.9 mg/mL and above (Mg10Gd, Pure Mg and Mg2Ag respectively) (Fig 5). The appearance of corrosion associated Mg at potentially cytotoxic concentrations (0.9–1.2 mg/mL) was, however, only observed after immersion of the Mg samples in the medium for at least 2 days (Fig 3). The cells, on the other hand, were only cultured for 24 hours on corroded samples in fresh medium. Mg ion release should, thereby, not have exceeded a concentration range of 0.5–0.6 mg/L during this period, i.e. concentrations that appeared to induce a significant increase in metabolic activity (Fig 5). Culturing of cells on Mg samples alone furthermore hampers Mg ion release. Mg ion concentration in the medium ranged from 0.2–0.5 mg/mL when MC3T3-E1 cells were cultured for 24 hours on non-corroded Pure Mg, Mg2Ag and Mg10Gd samples (S5 Fig). Release of Ag and Gd was at the limit of quantification (LOQ) during the first day of immersion, and time-dependent changes remained statistically non-significant (Table 3).

**Table 3:** Changes in Ag^+^ and Gd^3+^ release in Mg2Ag and Mg10Gd during 1, 2, 3 and 8 days of corrosion.

Mg2Ag	Day 1	Day 2	Day 3	Day 8
Ag^+^ (mg/mL)	LOQ	0.00145	0.002	0.002
Mg10Gd	Day 1	Day 2	Day 3	Day 8
Gd^3+^ (mg/mL)	LOQ	0.0007	0.0008	0.0012

Ag^+^ and Gd^3+^ release were measured in Mg2Ag and Mg10Gd immersed in DMEM supplemented with 10% FBS at 1, 2, 3 and 8 days with ICP-OES method. The amounts of ion released into the supernatant of both materials were low at all immersion times. LOQ were defined as a Limit of quantification.

The effect of pH on cell death can be excluded in this study, since the pH was adjusted to 7.4 in cell viability experiments (Fig 5). Furthermore, evaluation of cell viability without pH adjustment did not affect the cells as compared to the controls (cells cultured at 7.4) (S6 Fig).

These results indicate that the cell death observed during 24 hours culturing of MC3T3-E1 cells on Mg samples that have been pre-corroded for 2–3 days was not the result of cytotoxicity mediated by Mg ions released into the supernatant. Rather, the detrimental effect on cell viability appears to be closely linked to cell morphology (crystal formation) and/or the chemical surface content of corroded Mg samples (see Figs 1 and 2).

**Fig 6:**
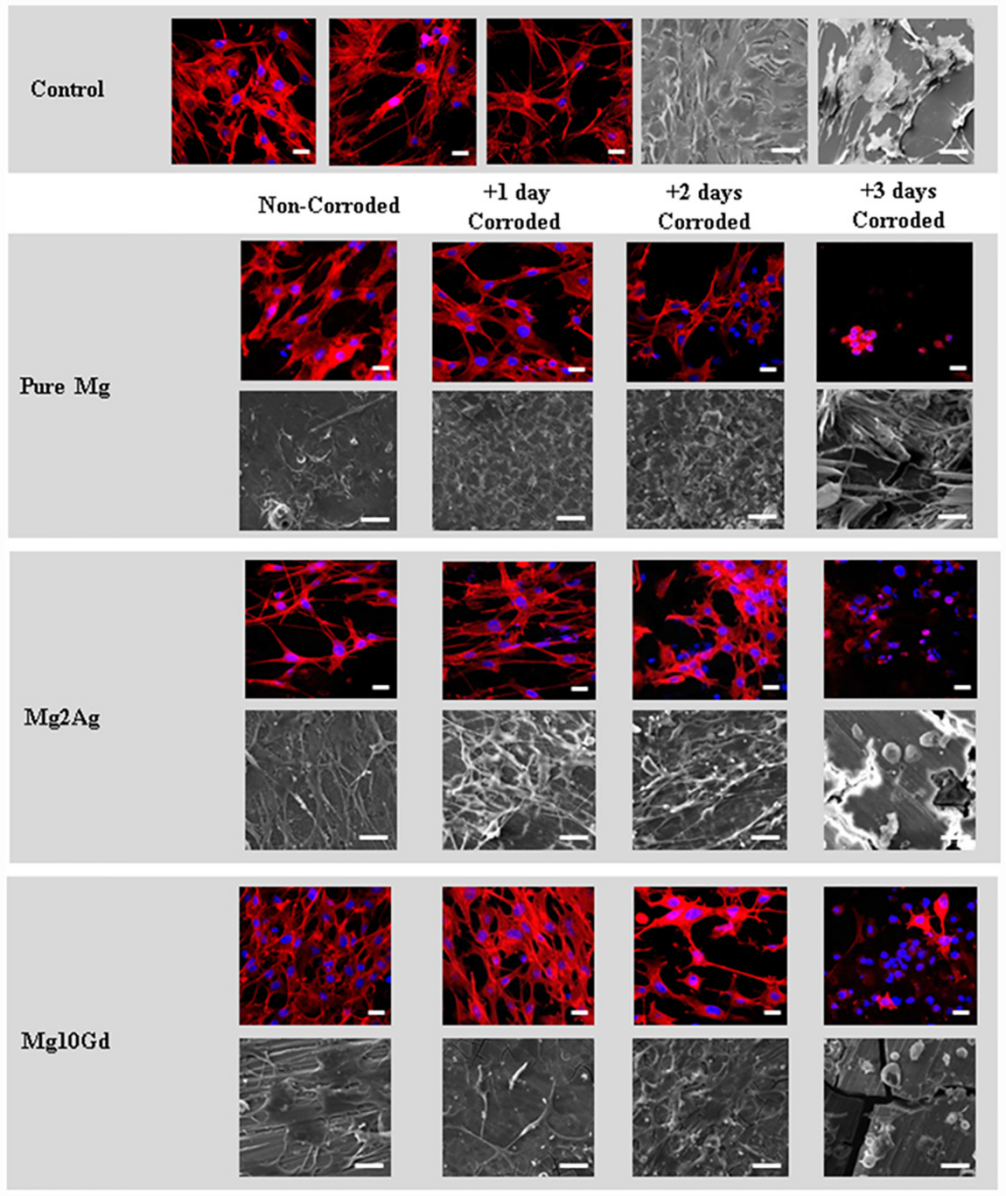
Changes in morphology of MC3T3-E1 cells cultured on corroded and non-corroded Pure Mg, Mg2Ag and Mg10Gd. MC3T3-E1 cells were cultured for 24hrs on non-corroded Pure Mg, Mg2Ag, Mg10Gd and samples which were corroded for 1, 2 and 3 days. Actin filaments were stained with Rhodamine-phalloidin and Cell nuclei were stained with DAPI for counterstaining. Images were merged at **40x**. Cells cultured on coverslips were used as a control. Moreover morphology of the cells was determined by scanning electron microscopy. **Scale bars**: 20 μm in florescent pictures and 30 μm in SEM pictures (n = 3 per group).

These suppositions are further supported by analysis of F-actin staining and electron microscopy of the changes in morphology of MC3T3-E1 cells cultured on corroded and non-corroded Mg samples. Differences in MC3T3-E1 cell morphology cultured on the distinct non-corroded Mg alloys, such as an increase in multiple cell processes when cells were grown on non-corroded Mg2Ag, and a flattening of cells following culturing on non-corroded Pure Mg or Mg10Gd, are indicative of a corrosion-independent effect of the sample surface on cell characteristics. However, corroded Mg alloys seemed to augment F-actin-based structuring of cells reminiscent of stress fibres, indicated also by an increase in cell extensions. This was particularly apparent when cells were grown on Pure Mg, Mg2Ag and Mg10Gd that had been pre-corroded for 1 to 2 days (Fig 6). Decreased numbers of cells were detected on all 3 day pre-corroded Mg samples. The few round cells detected on the surfaces indicated cell detachment, probably caused by cell death which accorded with the observed increase in cell death judged by viability testing of these samples (see Fig 4).

These observations indicate a detrimental effect of the surface of corroded Mg samples on cell characteristics such as viability and morphology. These effects appeared to be mediated mainly by the surface morphology and chemical surface content of the corroded Mg samples. Cell survival and cell morphologies indicating pronounced F-actin structuring of cells were observed only when cells were cultured on non-corroded Mg samples.

We subsequently analysed the potential of MC3T3-E1 cells to survive and differentiate during long term cultivation on non-corroded Mg samples.

### Effects of non-corroded Mg samples on cell MC3T3-E1 cell survival and differentiation potential

**Fig 7:**
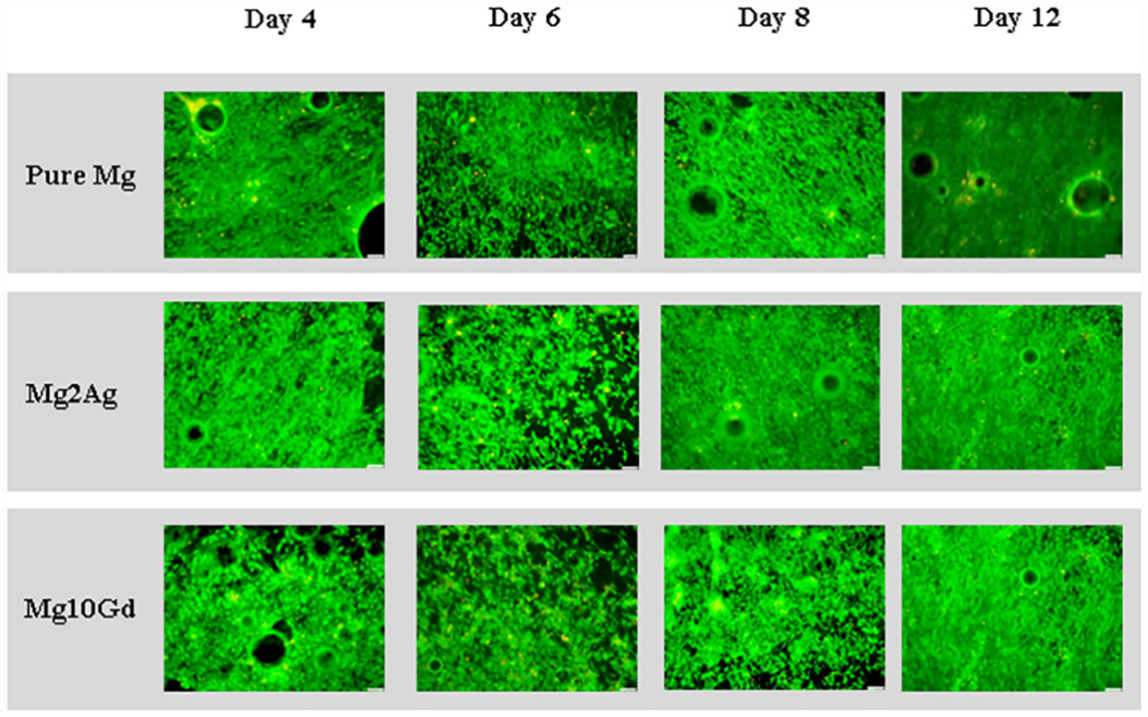
Viability of MC3T3-E1 cultured only on non-corroded Pure Mg, Mg2Ag and Mg10Gd for 4, 6, 8 and 12 days determined by live/dead staining. Viability of MC3T3-E1 cells was evaluated after 4, 6, 8 and 12 days of culture on non-corroded Pure Mg, Mg2Ag and Mg10Gd. **Scale bars**: 100 μm.

**Fig 8:**
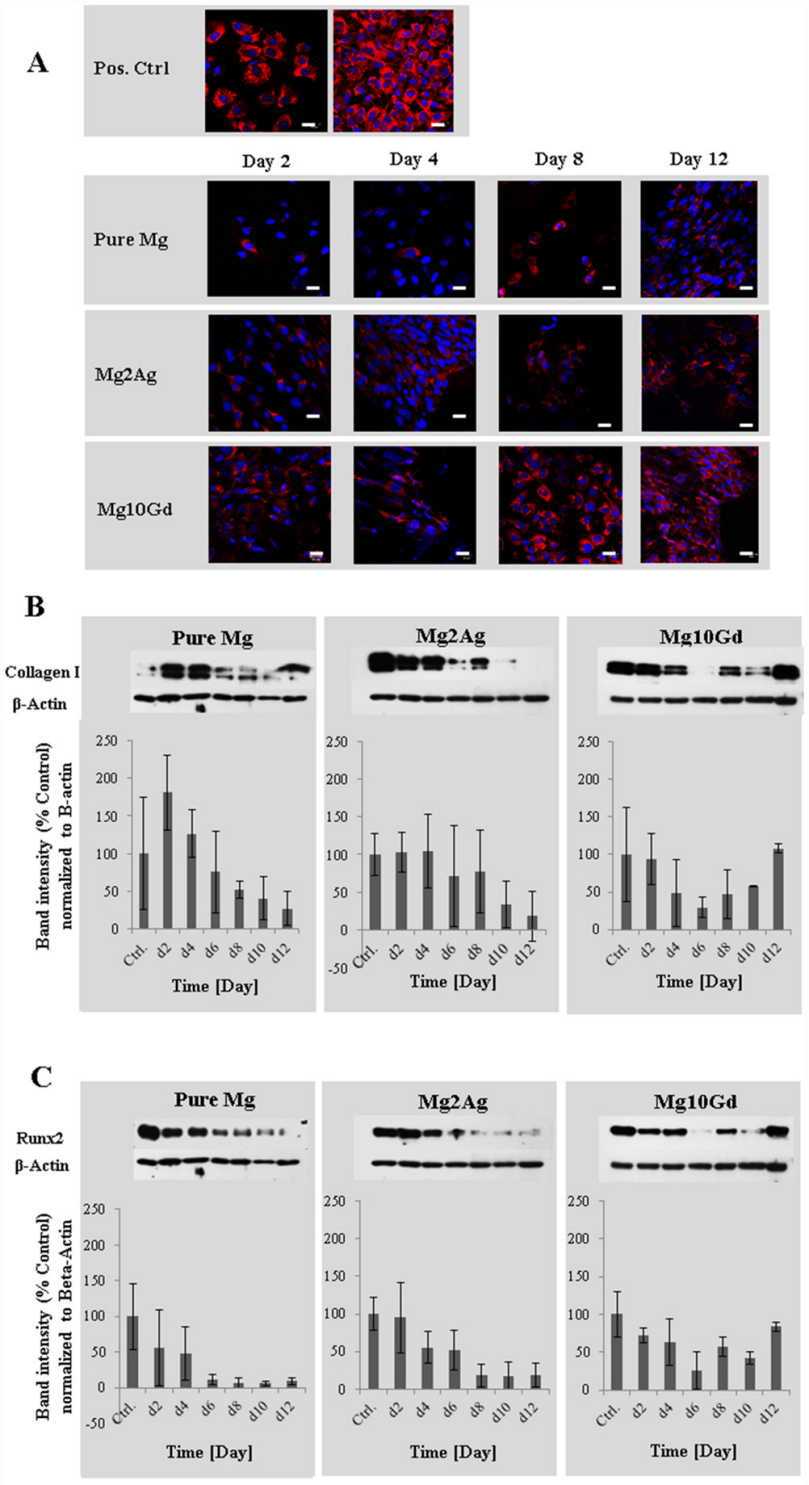
Expression of Collagen I and Runx2 in MC3T3-E1 cells which were cultured on non-corroded Mg and Mg alloys determined by Immunostaining and Western blot analysis. Immunostaining of MC3T3-E1 cells **(Panel A)** cultured on non-corroded Pure Mg, Mg2Ag and Mg10Gd for Collagen I at day 2, 4, 8 and 12. Cell nuclei were stained with DAPI for counterstaining. **Scale bars**: 20 μm. Protein expression levels of Collagen I and Runx2 in MC3T3-E1 cells **(Panel B and C)** cultured directly on non-corroded Pure Mg, Mg2Ag and Mg10Gd at day 2, 4, 6, 8, 10 and 12 determined by Western blot (n = 3 per group). The quantification values (Collagen I/B-Actin area and Runx2/ B-actin area) were verified by ImageJ software. The density of Collagen I and Runx2 were normalized to the B-Actin at each time point.

Culturing of MC3T3-E1 cells for 12 days on non-corroded Pure Mg, Mg2Ag and Mg10Gd did not result in any perceptible cell death assessed by Live/Dead staining (Fig 7). The osteogenic potential of MC3T3-E1 cells cultured on Mg based alloys, was determined by the measurement of Collagen I and Runx2 expression (Fig 8). An early, gradual, Mg alloy-independent down-regulation of Collagen I was observed by immunocytochemical and Western Blot analysis (Fig 8A and 8B). At late time points, however, a notable increase in Collagen I expression compared to control values (cells cultured on a tissue culture plate) was observed only when cells were cultured on Mg10Gd, indicating a recovery in osteogenic potential (Fig 8A and 8B). Collagen I expression by cells cultured on Pure Mg or Mg2Ag decreased during the entire culturing period. A similar expression pattern was observed for Runx2 by Western Blot analysis (Fig 8C). A recovery of Runx2 expression following initial down-regulation was only detected when cells were cultured on Mg10Gd. Runx2 expression by cells cultured on Pure Mg or Mg2Ag was continuously down-regulated, resulting in negligible expression levels after 12 days of culturing. Although pronounced, a statistically significant effect was not established. These results indicate a notable impairment of the cellular differentiation potential of MC3T3-E1 cells by Pure Mg and Mg2Ag.

## Discussion

Our study was primarily based on a clinical predicament, namely in vitro reports indicating that pre-corrosion of Mg-based materials before implantation might improve cell-material interaction and, thereby, subsequent tissue regeneration [52,65,66].

We therefore corroded Pure Mg as well as Mg2Ag and Mg10Gd (two promising alloys for orthopedic implant development) in cell culture medium and analysed the resultant alloy surfaces and their effects on cultured cells. Cell culture medium supports cell growth, but also contains a host of physiologically relevant substances, molecules and macromolecules, such as sugars, amino acids and proteins [67–69]. Culturing MC3T3-E1 cells in direct contact with corroded surfaces for 24 hr showed pre-corrosion of the Mg-based materials to significantly hamper cell survival. Cell death was closely linked to crystal formation on the corroded surfaces after 3 days of pre-corrosion, particularly on Pure Mg and Mg2Ag. Crystal formation was, in contrast, not observed on corroded Mg10Gd. EDX analysis linked crystal formation to an increase in atomic % oxygen, and a decrease in calcium and phosphorous content on Pure Mg and Mg2Ag that was not detected on crystal-free corroded Mg10Gd. Gd has been reported to be able to chelate both calcium and phosphor [63]. We therefore hypothesize that Gd chelated, and thereby surface bound calcium or phosphor, might slow oxidation and thereby suppress the formation of more stable crystals.

It should be noted that EDX analysis does not enable identification of the specific compounds that underlying corrosion deposits, films or crystal formation. It is, however, likely that substances such as Mg (OH)_2_, MgO, MgCO_3_, Mg_3_(PO_4_)_2_ and Ca_3_(PO_4_)_2_ are generated during the corrosion process in question, which is underlined by the high magnesium and oxygen surface contents [60]. There are, however, a number of other substances in the medium that could conceivably participate in the corrosion process, such as sugars, mirrored in the high carbon content of the corroded surfaces, and an undefined concentration of proteins (10% FBS) in particular [67–69]. How the generation of Mg-based corrosion deposits might vary when proteins with undefined charges are present and bind to the surfaces, remains to be studied.

Our results contradict a study reported by Willumeit et al [52]. Human osteosarcoma cells (Soas-2 cells) cultured on Pure Mg and Mg10Gd1Nd pre-corroded in DMEM supplemented with 10% FBS, exhibited higher cell viability than cells cultured on these materials without pre-corrosion. A formation of protective corrosion layers was proposed to explain this result. Notably though, crystal formation was not observed during corrosion in their study. Crystal formation on the surface of Pure Mg immersed for 72hr in DMEM supplemented with 10% FBS was, however, reported in an earlier publication [68], and crystal formation might, therefore, be a factor hampering cell survival. In the Willumeit et al. study, cells were cultivated on the corroded surfaces for 72 hr without a medium change, which might have resulted in increased Mg ion and pH values. We have shown that Mg ion release from Pure Mg enhances cell survival at low concentrations (0.2–0.8 mg/mL). This concentration range was detected when MC3T3-E1 cells were cultured on the surface of Pure Mg for up to three days, as per the Willumeit study. Other factors may, however, account for the observed discrepancy. Biocompatibility of non-corroded Pure Mg samples might be determined by the different Mg alloy, the production process and treatment of samples before corrosion, i.e. of sample surface sealing with NaOH, steam treatment or polishing [65,70–72]. Biocompatibility seems to be closely linked to the rate of corrosion of Mg samples, which might, in turn, also depend on several factors including the immersion system [73] stasis [13,69,74], non-stasis [13,75], the corrosion medium [67–69,76–78] and/or the associated pH [64,73,79]

An effect of pH on cell survival was excluded in our static corrosion system. The pH never exceeded 8.6 which had no effect on cell metabolism, in accordance with a report from Wang et al. indicating a stimulatory effect on growth of MC3T3-E1 and BMSC at a pH above 8.5 [80]. Toxic effects were observed when pH exceeded 8.6 [37,40,81]. Notably alkaline pH has also been shown to enhance crystal formation [82]. These pH effects might also explain some of the inconsistencies between corrosion studies.

Corrosion conditions aside, the comparison of different studies is also complicated by the use of different cell systems and cell lines [83]. This became apparent when the effect of Mg ion concentration on cell survival was analysed. While human bone osteosarcoma epithelial cells (U2-OS) appear to be inhibited by Mg concentrations of 5 mM and above [84], survival of MC3T3-E1 cells was hampered only at Mg ion concentrations greater than 12.5 mM. A similar Mg ion concentration cut-off point was reported for the SaOS-2, MG63, MC3T3-E1 and U2OS cell lines, HUVECs, primary human osteoblasts and chondrocytes [1,2,18,53,80,85–88]. Primary osteoblasts and the human osteosarcoma cell line (MG63) have been shown to be particularly resilient, with cell survival impaired at concentration above 59 mM Mg ions [85]. The loss of viability of different cell lines at different Mg ion concentrations might reflect the susceptibility of cells to corrosion products during cell material interaction.

We therefore concluded that surface morphology and content of corroded Pure Mg, Mg2Ag and Mg10Gd are the main factors determining cell survival in our system.

This was confirmed when MC3T3-E1 cells were cultured on non-corroded Pure Mg, Mg2Ag and Mg10Gd. Cells survived for 12 days and often reached confluence. This might be due, in part, to an inhibition of corrosion by the cells covering the surface, as demonstrated by a slower release of Mg ions and a slower pH increase in the medium in a study by Seuss F et al [37]. Differentiation potential, as determined by Runx2 and Collagen I expression, however decreased in a time-dependent manner when cells were cultured on Pure Mg and on Mg2Ag. Runx2 and Collagen I expression were up-regulated at late times points when cells were cultured on non-corroded Mg10Gd, indicating this alloy to be the more biocompatible material. Echoing our finding, Mg10Gd was deemed the most promising material by the Cecchinato study, due to its lower and homogeneous initial corrosion rate, and impressive biocompatibility properties [39].

## Conclusion

Summing up, we therefore conclude that the corroded surfaces of Pure Mg, Mg2Ag and Mg10Gd negatively impact MC3T3-E1 cell viability under near physiological conditions. Our results indicate that cell viability might be hampered by corrosion associated crystal formation in particular, and to some extent by surface content. Our studies further revealed that low concentrations of Mg ions and pH values up to pH 8.6, might support cell survival. This is important information for clinical approaches since increases in Mg ion concentration and pH values might be expected at the site of implantation. Our results showed non-corroded Mg10Gd to be the most biocompatible material in our study, whilst corroded Pure Mg was the least favourable material for MC3T3-E1 cell survival. Mg10Gd thus appears to possess suitable properties for orthopaedic applications.

## Supporting Information

S1 Fig Surface topography and roughness parameters of Pure Mg, Mg2Ag and Mg10Gd discs. Roughness of each alloy were measured by a Contour GT—K Bruker Profilometer using white light interferometry (A) and surface roughness of all three alloys were compared to each other (B).
(TIF)

S2 Fig Microstructure of Pure Mg, Mg2Ag and Mg10Gd. Microstructures were observed using an optical microscope with a digital camera. The grain size was determined using the line intercept method to calculate the middle square grain diameter. Mg2Ag and Pure Mg generated similar grain sizes, whilst Mg10Gd showed smaller middle square grain diameter.
(TIF)

S3 Fig Elemental composition of Mg and Mg alloys quantified by EDS mapping, independent of time points based on structure of corroded regions. Mg and Mg alloys immersed in cell culture medium were analysed by scanning electron microscopy equipped with Energy-dispersive X-ray spectroscopy (EDS). Measurements were done according to the changes in surface structure (light resembling protruding regions, dark areas and needle shape crystals) during immersion period in DMEM with 10% FBS independent of time points. Elemental composition was calculated based on atomic percentage of corroded regions.
(TIF)

S4 Fig Changes in the pH value of Pure Mg, Mg2Ag and Mg10Gd during 1, 2, 3 and 8 days of immersion in DMEM supplemented with 10%FBS. (TIF)

S5 Fig Changes in Mg^2+^ release in Pure Mg, Mg2Ag and Mg10Gd when MC3T3-E1 cells were cultivated on the surface. The Mg ion release was measured during culturing of MC3T3-E1 cells on the surface of the non-corroded Mg and Mg alloys for 1, 2 and 3 days by ICP-OES; n = 5. Statistical significance was tested with One-Way ANOVA test. **#p<0.05** as compared to the control (Magnesium level of the basal medium).
(TIF)

S6 Fig Viability of MC3T3-E1 cells treated with different concentration of Mg^2+^ derived from Pure Mg, Mg2Ag and Mg10Gd extracts determined by MTT assay. Viability of MC3T3-E1 cells determined by MTT assay after incubation for 24hrs with 0.3, 0.6, 0.9 and 1.2 mg/ml Mg^2+^ resulted from Pure Magnesium, Mg2Ag and Mg10Gd extracts. The pH of the extracts did not adjust to physiological level. At pH of 8.6 cells viability was not affected. Statistical significance was tested with One-way ANOVA test. * p<0.05 as compared to cell viability of the control; **# p<0.05** as compared to cell viability at concentration of 1.2 mg/ml Mg^2+^ derived from Pure Mg extracts; **§ and°: p<0.05** as compared to cell viability at concentrations of 1.2 mg/ml Mg^2+^ derived from Mg2Ag extracts.
(TIF)
