# Supplementary figures and images for: Effects of Corroded and Non-Corroded Biodegradable Mg and Mg Alloys on Viability, Morphology and Differentiation of MC3T3-E1 Cells Elicited by Direct Cell/Material Interaction
Source: PLoS One. 2016 Jul 26;11(7):e0159879. doi: 10.1371/journal.pone.0159879 (PMC4961286; doi:10.1371/journal.pone.0159879)

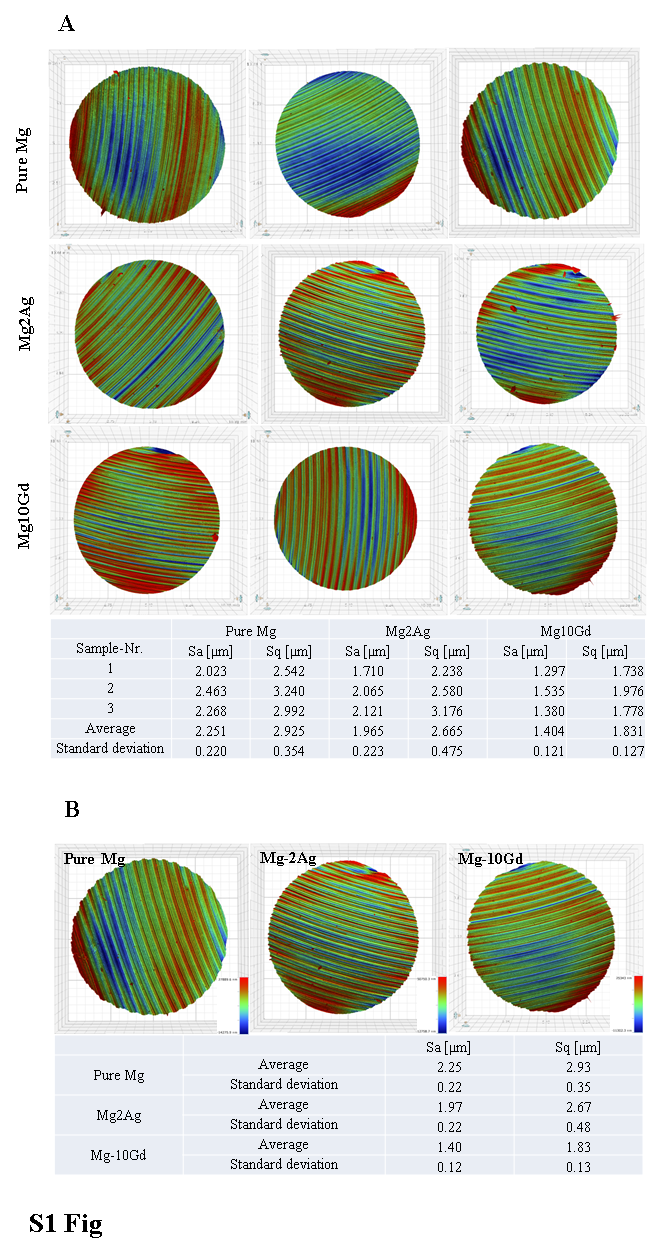

Supplement: S1 Fig — Roughness of each alloy were measured by a Contour GT—K Bruker Profilometer using white light interferometry (A) and surface roughness of all three alloys were compared to each other (B). (TIF) [file pone.0159879.s001.tif]

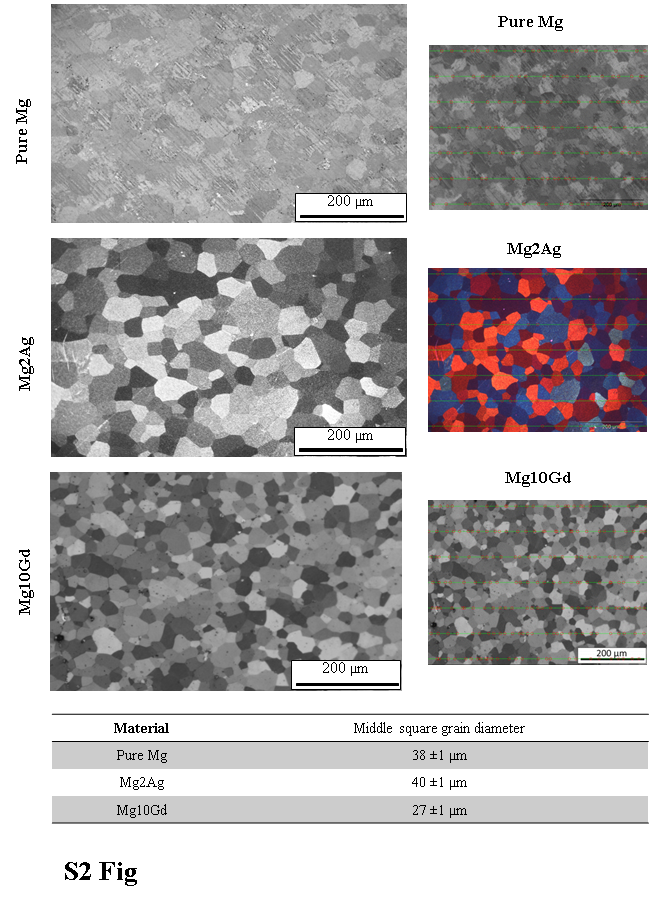

Supplement: S2 Fig — Microstructures were observed using an optical microscope with a digital camera. The grain size was determined using the line intercept method to calculate the middle square grain diameter. Mg2Ag and Pure Mg generated similar grain sizes, whilst Mg10Gd showed smaller middle square grain diameter. (TIF) [file pone.0159879.s002.tif]

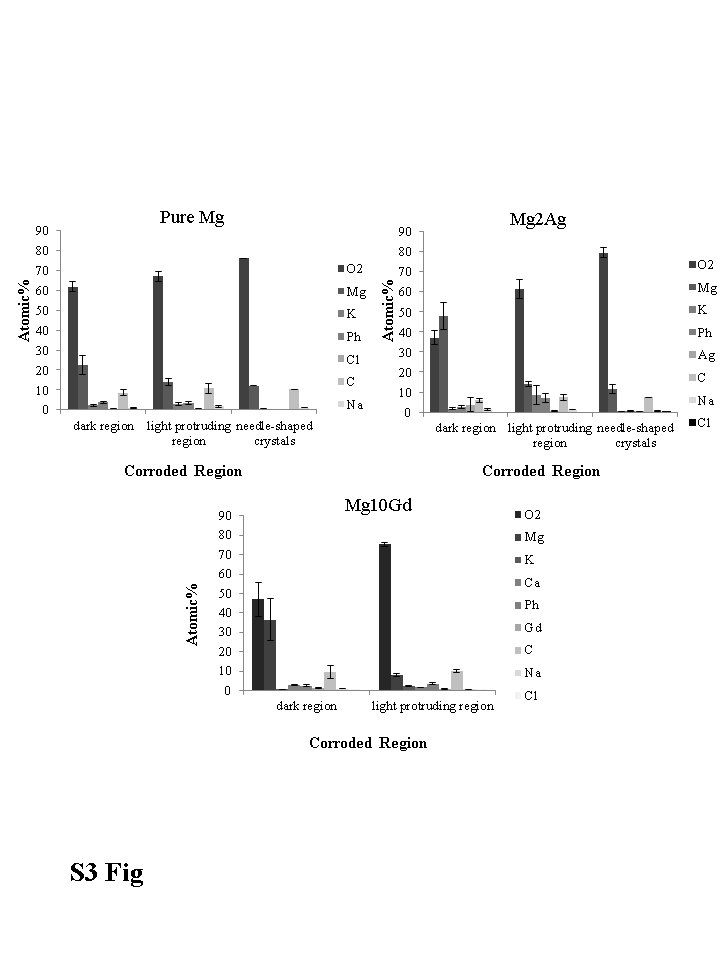

Supplement: S3 Fig — Mg and Mg alloys immersed in cell culture medium were analysed by scanning electron microscopy equipped with Energy-dispersive X-ray spectroscopy (EDS). Measurements were done according to the changes in surface structure (light resembling protruding regions, dark areas and needle shape crystals) during immersion period in DMEM with 10% FBS independent of time points. Elemental composition was calculated based on atomic percentage of corroded regions. (TIF) [file pone.0159879.s003.tif]

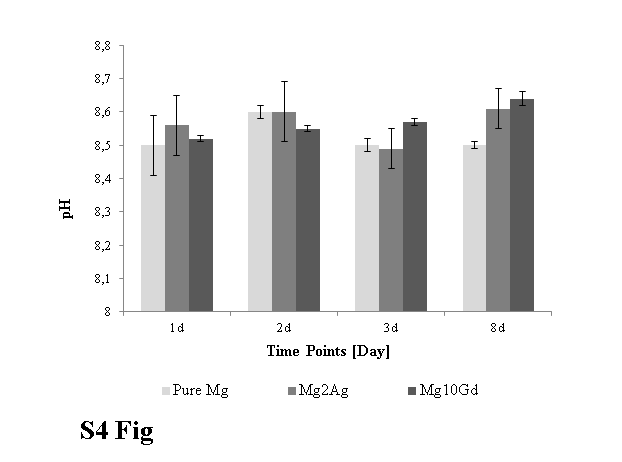

Supplement: S4 Fig — (TIF) [file pone.0159879.s004.tif]

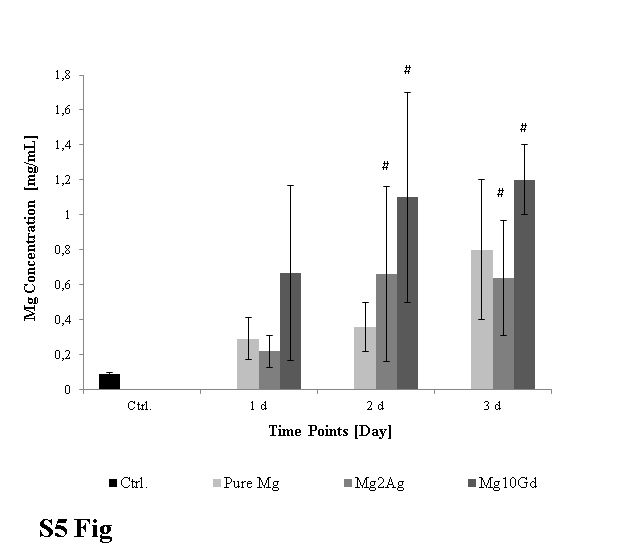

Supplement: S5 Fig — The Mg ion release was measured during culturing of MC3T3-E1 cells on the surface of the non-corroded Mg and Mg alloys for 1, 2 and 3 days by ICP-OES; n = 5. Statistical significance was tested with One-Way ANOVA test. #p<0.05 as compared to the control (Magnesium level of the basal medium). (TIF) [file pone.0159879.s005.tif]

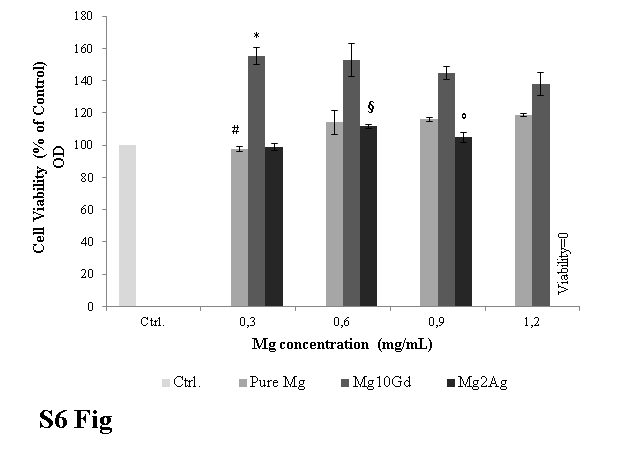

Supplement: S6 Fig — Viability of MC3T3-E1 cells determined by MTT assay after incubation for 24hrs with 0.3, 0.6, 0.9 and 1.2 mg/ml Mg2+ resulted from Pure Magnesium, Mg2Ag and Mg10Gd extracts. The pH of the extracts did not adjust to physiological level. At pH of 8.6 cells viability was not affected. Statistical significance was tested with One-way ANOVA test. * p<0.05 as compared to cell viability of the control; # p<0.05 as compared to cell viability at concentration of 1.2 mg/ml Mg2+ derived from Pure Mg extracts; § and°: p<0.05 as compared to cell viability at concentrations of 1.2 mg/ml Mg2+ derived from Mg2Ag extracts. (TIF) [file pone.0159879.s006.tif]
